# Enumeration of *Mycobacterium avium *subsp. *paratuberculosis *by quantitative real-time PCR, culture on solid media and optical densitometry

**DOI:** 10.1186/1756-0500-5-114

**Published:** 2012-02-22

**Authors:** Petr Kralik, Vladimir Beran, Ivo Pavlik

**Affiliations:** 1 Veterinary Research Institute, Hudcova 70, 621 00 Brno, Czech Republic

## Abstract

**Background:**

Different approaches are used for determining the number of *Mycobacterium avium *subsp. *paratuberculosis *(MAP) cells in a suspension. The majority of them are based upon culture (determination of CFU) or visual/instrumental direct counting of MAP cells. In this study, we have compared the culture method with a previously published F57 based quantitative real-time PCR (F57qPCR) method, to determine their relative abilities to count the number of three different MAP isolates in suspensions with the same optical densities (OD). McFarland turbidity standards were also compared with F57qPCR and culture, due to its frequent inclusion and use in MAP studies.

**Findings:**

The numbers of MAP in two-fold serial dilutions of isolates with respective OD measurements were determined by F57qPCR and culture. It was found that culture provided lower MAP CFU counts by approximately two log_10_, compared to F57qPCR. The McFarland standards (as defined for *E. coli*) showed an almost perfect fit with the enumeration of MAP performed by F57qPCR.

**Conclusions:**

It is recommended to use culture and/or qPCR estimations of MAP numbers in experiments where all subsequent counts are performed using the same method. It is certainly not recommended the use of culture as the standard for qPCR experiments and *vice versa*.

## Findings

The sensitivity of detection of *Mycobacterium avium *subsp. *paratuberculosis *(MAP) in different matrices is linked to the number of bacteria present. However, determining the exact number of MAP cells in a sample is complicated with no real consensus on an approved method within the scientific community. Several methods have been suggested for the enumeration of MAP in routine diagnostics and laboratory experiments. Culture on solid media with subsequent counting of colony forming units (CFU) is the most widely used method [1]. Unfortunately, a major problem encountered with this method is the long incubation period required to grow MAP as well as its tendency to form clumps when culturing in broth *in vitro*. Despite this, culture is presently the only method that can provide quantitative data on the number of viable MAP cells in a sample. It is used as the quantification standard for the optimization of PCR methods and DNA isolation procedures from milk and faeces [2,3].

Another possible method how to assess the number of MAP cells in a sample is visual or instrumental counting of individual cells. Visual counting of MAP cells under a microscope can serve as a confirmatory method to culture [3,4]. Instrumental counting of MAP cells can be performed using a haemocytometer or flowcytometer [5,6]. MAP can also be successfully enumerated by the quantitative real-time PCR (qPCR) method based upon the single copy fragment F57 [7,8]. Compared to culture, these counting methods assess the number of MAP cells independently from their viability.

In order to assess the approximate number of bacteria in a sample quickly and easily, turbidity measurements were introduced. Optical densities (OD) of the bacterial suspensions were determined at wavelengths between the range 550 to 600 nm, which corresponds to the bacterial absorption maximum of the sample [9]. For MAP there have been several equations used to calculate the number of MAP cells from OD readings. Janagama et al. (2006) stated that OD_600 nm _= 0.3 is equal to 10^9 ^MAP CFU/ml of suspension [10]. Bogli-Stuber et al. (2005) counted MAP according to the equation OD_540 nm _= 0.65 corresponds to 4 × 10^8 ^CFU/ml [11]. In contrast, Chui et al. (2004) used the formula OD_550 nm _= 1 is equivalent to 2.8 × 10^6^-10^7 ^MAP cells/ml of suspension [12].

An alternative variant of turbidity measurements are the McFarland standards [13]. The bacterial suspensions are then visually compared to the McFarland standards estimating the bacterial density. For *E. coli*, a 0.5 McFarland standard corresponds to 1.5 × 10^8 ^CFU/ml [9]. This technique is widely used in microbiology and has been adopted for MAP [5,14].

The heterogeneity of MAP enumeration using OD measurements, combined with the lack of direct comparison with culture and individual cell counting methods brings forward the additional problem of interpreting results from different laboratories. The aim of this study was to compare two distinct methods of MAP quantification, culturing on solid media and F57qPCR, for their ability to quantify the number of MAP cells in serially diluted aliquots of three different MAP isolates with identical OD_600 nm _readings. In accordance with routine laboratory practice, McFarland turbidity standards were prepared, their exact OD were determined and the theoretical amount of bacterial cells [9] was compared with F57qPCR and culture results to assess its applicability for the enumeration of MAP.

## Materials and methods

### Preparation of MAP isolates

All three MAP isolates used in this study were obtained from cow faeces and tissues at the Veterinary Research Institute (Brno, Czech Republic) and belonged to the RFLP type C1. Isolates 8819 and 8672 were both passaged five times and produced visible colonies within 8 weeks on Herrold's Egg Yolk Medium (HEYM) with Mycobactin J (Allied Monitor, Fayette, MO, USA) with the antibiotics penicillin G, chloramphenicol and amphotericin B. Isolate 12146 was passaged more than 10 times and produced colonies on HEYM with supplements in under 6 weeks. A single colony from each isolate was inoculated into liquid Middlebrook 7H9 broth (DIFCO, Livonia, MI, USA), supplemented with Middlebrook OADC enrichment (DIFCO) and Mycobactin J (Allied Monitor) and then cultured for up to 5 weeks at 37°C, to avoid excessive cell clumping.

Aliquots of 50 ml from each MAP isolate in liquid medium were centrifuged at 7 000 g for 2 min, subsequently discarding the supernatant. The pellets were resuspended in the remaining supernatant (approximately 1 ml) and transferred to 2 ml screw cap tubes, each containing twelve 1 mm zirconia silica beads (Biospec, Bartlesville, OK, USA), vortexed at full speed for 10 s and centrifuged at 100 g for 30 s. These steps were carried out to minimize the number of MAP clumps in cell suspensions. The presence of MAP clumps in each MAP suspension was checked using Ziehl-Neelsen staining and optical microscopy.

### Optical density determination

Each suspension of MAP isolate was two-fold serially diluted in Middlebrook 7H9 medium in seven succeeding steps to ensure that zero OD was reached. Each dilution was split into three aliquots of 500 μl and in portion of each of them (60 μl) absorbance at 600 nm was recorded (Biophotometer, Eppendorf, Hamburg, Germany). The remainder of the aliquots was used for F57qPCR and culture. Middlebrook 7H9 medium was used as the blank.

### Determination of the absolute number of MAP in diluted suspensions by F57qPCR

Three aliquots of 200 μl from the two-fold diluted MAP suspensions were centrifuged at 7 000 g for 2 min, the supernatant was removed by pipetting and the pellet was resuspended in 300 μl of Tris-EDTA (TE) buffer supplemented with Fish Sperm DNA (Serva, Heidelberg, Germany) at a concentration of 50 ng/μl. After the addition of 350 mg of 0.1 mm zirconia silica beads (Biospec) the MAP cells were lysed using a MagNA Lyser (Roche Molecular Diagnostic, Manheim, Germany) at 6 400 rpm for 60 s. The lysed MAP cells were centrifuged at 18 000 g for 5 min and the supernatant was then used as the template for qPCR, amplifying the single copy fragment *F57 *[7]. The absolute quantity of MAP cells was determined according to the calibration curve, derived from 10-fold dilutions of plasmid standards containing the F57qPCR product insert in the range from 5 × 10^5 ^to 5 × 10^0 ^copies per F57qPCR reaction [7].

### Determination of MAP CFU counts in diluted suspensions by solid culture

Three aliquots from each serial two-fold dilution were immediately after OD determination diluted 1:100 in Middlebrook 7H9 in two consecutive steps for the purpose of CFU number determination by culture. One hundred micro litres of the undiluted and diluted solutions (1:100 and 1:10 000) were precisely spread on HEYM with Mycobactin J and antibiotics and incubated at 37°C for 3 months.

### Comparison of F57qPCR and culture with statistical evaluation of the results

Mean OD values of each isolate suspension were calculated from the triplicate aliquots prepared for the determination of absorbance. These values were plotted against the log_10 _of the mean absolute numbers of MAP and CFU gained by F57qPCR and culture (semi-logarithmic plot), respectively. The linearity of F57qPCR and culture methods was checked by calculating the square Spearman's rank correlation coefficient (R^2^). To compare the MAP numbers gained by F57qPCR and culture with McFarland turbidity standards, standards of 0.5, 1 and 2 McFarland were included in the plot. The optical density of each McFarland standard was measured and paired with an estimated bacterial cell density (number of CFU of *E. coli*) according to the following approximations: McFarland 0.5 = 1.5 × 10^8 ^CFU/ml, McFarland 1 = 3.0 × 10^8 ^CFU/ml and McFarland 2 = 6.0 × 10^8 ^CFU/ml [9].

Because it was not possible to asses normality of data from triplicates Mann-Whitney Test was used to evaluate statistically results gained by F57qPCR and culture for the relevant dilution and isolate. *P*-values lower than 0.05 were considered statistically significant. The differences in logarithms of absolute counts between F57qPCR and culture data were expressed as logarithm of quotient of mean absolute counts from F57qPCR and mean CFU counts from culture.

## Results and discussion

Despite all attempts to reduce the number of MAP clumps, in all three MAP isolates there were still several units or even tens of small clumps present, visible after staining with Ziehl-Neelsen and optical microscopy. From this, we can conclude that it is very difficult to get rid of all MAP clumps in a suspension making data from culture and qPCR incomparable.

**Figure 1:**
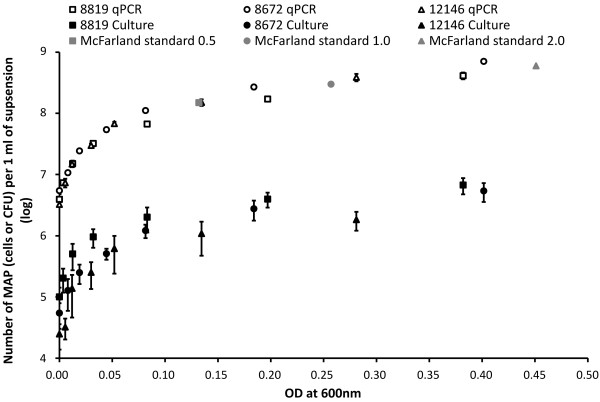
**Semilogarithmic graph comparing enumeration of three *Mycobacterium avium *subsp. *paratuberculosis *(MAP) isolates**. White symbols represent absolute MAP numbers of three MAP isolates gained by F57 based qPCR for the respective OD and dark symbols represent CFU numbers gained by culture. Error bars represent standard deviations obtained from three independent physical replicates. McFarland standards 0.5, 1 and 2 are shown by grey symbols. The OD of each McFarland standard was determined in this study; theoretical number of bacterial cells was adopted according to the standard formula for *E. coli*: McFarland 0.5 = 1.5 × 10^8 ^CFU/ml [9].

The absolute numbers of MAP determined by F57qPCR were approximately two log_10 _greater than the CFU counts from culture at respective ODs (Figure 1) and a highly significant statistical difference (*P *< 0.0001) between all the compared samples was observed. This trend was characteristic for all three MAP isolates used in the study allowing us to exclude any possible differences in the *in vitro *viability of the cells at the isolate level. Moreover, it is in concordance with previous observations when quantification methods that do not distinguish between viable and dead cells (visual and instrumental counting and qPCR) provide higher absolute numbers of MAP compared to CFU counts using the culture method [5,7].

The mean differences between F57qPCR and culture data was 1.58 for isolate 8819, 2.00 for isolate 8672 and 2.13 for isolate 12146 in log_10_. The difference between F57qPCR and culture method can be explained by the formation of MAP clumps and the viability of MAP. It was previously hinted that MAP counting using the culture method could be affected by cell clumping, which can lead to an underestimation of MAP cells due to CFU's arising from more than 1 cell. This hypothesis is supported by the fact that colonies on the same plate grow at different rates and are different sizes [15].

The other major factor influencing the discrepancy in results between the two methods is visualisation of both viable and non-viable cells. Due to the fact that culture can visualise viable *MAP *cells only, the reduction of culture counts can be assumed. A MAP suspension, which should correspond to McFarland 10^9 ^CFU/ml, was determined to contain only 2.3 × 10^8 ^CFU/ml [16].

The dependence of OD and F57qPCR or CFU numbers was shown to be linear and all square Spearman's rank correlation coefficients were close to 1 (Table 1). The limit of detection for OD MAP enumeration by F57qPCR was similar for all isolates and reached approximately 4 × 10^6 ^MAP cells per ml, whereas for culture it was lower at 3 × 10^4 ^CFU/ml (Table 1).

**Table 1:** Comparison of recovery of *Mycobacterium avium *subsp. *paratuberculosis *by real-time PCR and culture

Isolate	OD*^a^*	F57qPCR			Culture			Difference*^f^*
		Copies/ml*^b^*	SD*^c^*	SC*^d^*	CFU/ml*^e^*	SD*^c^*	SC*^d^*	
8819	0.382	4.13 × 10^8^	5.24 × 10^7^	0.972	6.77 × 10^6^	2.01 × 10^6^	0.994	1.79
	0.197	1.71 × 10^8^	5.43 × 10^6^		3.98 × 10^6^	1.10 × 10^6^		1.63
	0.083	6.69 × 10^7^	5.73 × 10^6^		2.02 × 10^6^	8.84 × 10^5^		1.52
	0.032	3.22 × 10^7^	3.27 × 10^6^		9.58 × 10^5^	3.23 × 10^5^		1.53
	0.013	1.51 × 10^7^	1.82 × 10^6^		5.05 × 10^5^	2.32 × 10^5^		1.47
	0.004	7.35 × 10^6^	4.78 × 10^5^		2.03 × 10^5^	8.59 × 10^4^		1.56
	0.000	3.94 × 10^6^	8.55 × 10^5^		1.00 × 10^5^	4.14 × 10^4^		1.59

8672	0.402	7.09 × 10^8^	1.84 × 10^7^	0.998	5.42 × 10^6^	1.85 × 10^6^	0.990	2.12
	0.184	2.70 × 10^8^	2.30 × 10^7^		2.77 × 10^6^	9.99 × 10^5^		1.99
	0.082	1.11 × 10^8^	5.42 × 10^6^		1.22 × 10^6^	3.01 × 10^5^		1.96
	0.045	5.42 × 10^7^	4.58 × 10^6^		5.10 × 10^5^	1.04 × 10^5^		2.03
	0.019	2.44 × 10^7^	1.25 × 10^6^		2.50 × 10^5^	8.75 × 10^4^		1.99
	0.008	1.07 × 10^7^	9.01 × 10^5^		1.28 × 10^5^	6.84 × 10^4^		1.92
	0.000	5.48 × 10^6^	5.29 × 10^5^		5.45 × 10^4^	2.44 × 10^4^		2.00

12146	0.281	3.89 × 10^8^	5.45 × 10^7^	0.989	1.83 × 10^6^	6.25 × 10^5^	0.968	2.33
	0.135	1.51 × 10^8^	1.86 × 10^7^		1.09 × 10^6^	6.15 × 10^5^		2.14
	0.052	6.80 × 10^7^	4.95 × 10^6^		6.17 × 10^5^	3.76 × 10^5^		2.04
	0.030	2.99 × 10^7^	2.02 × 10^6^		2.52 × 10^5^	1.17 × 10^5^		2.07
	0.012	1.48 × 10^7^	1.64 × 10^6^		1.38 × 10^5^	9.16 × 10^4^		2.03
	0.006	7.37 × 10^6^	1.20 × 10^6^		3.22 × 10^4^	1.20 × 10^4^		2.36
	0.000	3.26 × 10^6^	2.87 × 10^5^		2.48 × 10^4^	1.10 × 10^4^		2.12

qPCR: quantitative real-time PCR

*^a ^*OD: optical density at 600 nm, values represent mean of three independent measurements

*^b ^*Mean number of F57 copies (which is equal to number of *Mycobacterium avium *subsp. *paratuberculosis *cells) from a triplicate in a respective dilution

*^c ^*Standard deviation

*^d ^*Square Spearman's rank correlation coefficient (R^2^) expressing linear dependence of F57qPCR (or culture) counts with OD

*^e ^*Mean number of CFU recovered by culture on solid media from triplicate in a respective dilution

*^f ^*Difference in log_10 _between mean value gained by F57qPCR and culture expressed as log_10_(copies/ml) - log_10_(CFU/ml)

MAP quantification using the culture method therefore cannot be used as the reference method for qPCR and *vice versa*. It is necessary to choose a suitable quantification method with respect to the application of experimental data. This must be taken into account particularly when spiking food or faecal matrices with MAP to determine the limit of detection (LOD) of PCR and qPCR methods. According to the results of this study, using such "inaccurate" MAP number determination methods can lead to gross underestimations of the LOD for the respective PCR or qPCR system [2,3,11,17].

This study provides a model for estimating the number of MAP cells in liquid media, through measuring its OD. According to Table 1 and Figure 1, one can use OD measurements to assess quickly an approximate number (CFU) of MAP in a sample. This model is flexible because it does not require the dilution of the sample to an exact OD, as is required using the fixed equations [10-12] or McFarland standards. Perfect fits of F57qPCR MAP numbers and theoretical CFUs of *E. coli *at respective ODs strengthen the reliability of F57qPCR enumeration and highlight the problems with quantification of MAP by culture (Figure 1). However, if the model were to be functional, samples would have to be prepared identically to the procedure described in the Materials and Methods. Failure to do so is likely to lead to a gross underestimation of MAP numbers or CFUs, due to MAP cells tendency to form clumps.

In summary, it was concluded that using the culture method for MAP enumeration provides two log_10 _lower counts compared to qPCR. This was shown using three different MAP isolates. Possible reasons for this include the presence of clumps in the suspension and the omission of non-viable cells. MAP counts obtained by qPCR are not influenced by the presence of clumps or the viability of MAP. Based on data, it is recommended using culture and/or qPCR estimations of MAP numbers in experiments when all subsequent counts are performed using the same method(s). It is definitely not recommended to use culture as the standard for qPCR experiments and *vice versa*.

## Abbreviations

CFU: Colony forming unit; MAP: *Mycobacterium avium *subsp. *paratuberculosis; *OD: Optical density; qPCR: Real-time quantitative polymerase chain reaction; RFLP: Restriction fragment length polymorphism.

## Competing interests

The authors declare that they have no competing interests.

## Authors' contributions

PK conceived the whole study and performed measurement of OD and quantification of MAP by qPCR and drafted the manuscript. VB performed the culture experiments and determination of MAP CFU. IP participated on the scientific design of the study. All authors participated intellectually on the writing of this manuscript. All authors read and approved the final manuscript.
